# Risk factors of non-sentinel lymph node metastasis in breast cancer with 1–2 sentinel lymph node macrometastases underwent total mastectomy: a case-control study

**DOI:** 10.1186/s12957-023-02888-z

**Published:** 2023-04-06

**Authors:** Zhen Huang, Zhe Wu, Quan-qing Zou, Yu-jie Xie, Li-hui Li, Yan-ping Huang, Feng-ming Wu, Dong Huang, Yin-hua Pan, Jian-rong Yang

**Affiliations:** 1 Department of Breast Surgery, People’s Hospital of Guangxi Zhuang Autonomous Region, Guangxi Academy of Medical Sciences, No. 6 Taoyuan Road, Nanning, 53002 China; 2 Department of Gynaecology, People’s Hospital of Guangxi Zhuang Autonomous Region, Guangxi Academy of Medical Sciences, Nanning, China; 3 Department of Breast Surgery, Fourth Affiliated Hospital of Guangxi Medical University, Liuzhou, China

**Keywords:** Breast cancer, Total mastectomy, Sentinel lymph node, Macrometastasis, Non-sentinel lymph node, Risk factor

## Abstract

**Background:**

The randomized trials which include ACOSOG Z0011 and IBCSG 23-01 had found that the survival rates were not different in patients with cT1/2N0 and 1–2 sentinel lymph node (SLN)-positive, macro/micrometastases who underwent breast-conserving therapy, and micrometastases who underwent total mastectomy (TM), when axillary lymph node dissection (ALND) was omitted. However, for patients with cT1/2N0 and 1–2 SLN macrometastases who underwent TM; there was still insufficient evidence from clinical studies to support whether ALND can be exempted. This study aimed to investigate the risk factors of non-sentinel lymph node (nSLN) metastasis in breast cancer patients with 1–2 SLN macrometastases undergoing TM.

**Methods:**

The clinicopathological data of 1491 breast cancer patients who underwent TM and SLNB from January 2017 to February 2022 were retrospectively analyzed. Univariate and multivariate analyses were performed to analyze the risk factors for nSLN metastasis.

**Results:**

A total of 273 patients with 1–2 SLN macrometastases who underwent TM were enrolled. Postoperative pathological data showed that 35.2% patients had nSLN metastasis. The results of multivariate analysis indicated that tumor size (TS) (*P* = 0.002; OR: 1.051; 95% CI: 1.019–1.084) and ratio of SLN macrometastases (*P* = 0.0001; OR: 12.597: 95% CI: 4.302–36.890) were the independent risk factors for nSLN metastasis in breast cancer patients with 1–2 SLN macrometastases that underwent TM. The ROC curve analysis suggested that when TS ≤22 mm and ratio of SLN macrometastases ≤0.33, the incidence of nSLN metastasis could be reduced to 17.1%.

**Conclusions:**

The breast cancer patients with cT1/2N0 stage, undergoing TM and 1–2 SLN macrometastases, when the TS ≤22 mm and macrometastatic SLN does not exceed 1/3 of the total number of detected SLN, the incidence of nSLN metastasis is significantly reduced, but whether ALND can be exempted needs further exploration.

## Background

Breast cancer has recently risen in prevalence, making it one of the most prevalent malignant tumors hazardous to women’s health. Axillary lymph node dissection (ALND) used to be the standard treatment of axillary surgery for early breast cancer. However, the complications such as upper limb motor dysfunction, lymphedema, and sensory loss that may be caused by ALND adversely impact nearly 39% of postoperative patients [1].

With the application of sentinel lymph node biopsy (SLNB) and its support by safety studies, the surgical management of the axillary in breast cancer patients has undergone significant changes [2]. For patients with preoperative cN+, ALND is still recommended. However, for cN0 patients, SLNB should be recommended whenever possible to reduce the risk of postoperative complications, after understanding the safety and possible risks of SLNB. Among patients with positive sentinel lymph node (SLN), according to the ACOSOG Z0011 study, it is safe and feasible to avoid ALND in patients with cT1/2N0 and 1–2 SLN-positive (macro/micrometastases) who received breast-conserving therapy (BCT) for up-front surgery (exclusion of surgery after neo-adjuvant chemotherapy) [3]. According to the findings of the IBCSG 23-01 trial, patients who underwent total mastectomy (TM) and had cT1/2N0 and 1–2 SLN micrometastases may also be eligible for an ALND exemption, but there were not many cases of total mastectomy [4]. For patients with cT1/2N0 and 1–2 SLN macrometastases who underwent TM, the EORTC AMAROS study supports the safe and feasible replacement of ALND by axillary radiotherapy (ART). The authors reported that despite this, non-inefficiencies were not achieved due to an insufficient number of patients [5]. However, there is still insufficient evidence from clinical studies to support whether further axillary treatment including ALND or ART can be safely exempted.

In previous studies, a variety of clinicopathological factors were considered to be associated with non-sentinel lymph node (nSLN) metastasis in SLN-positive breast cancer [6–11]. This study will further explore the risk factors for the development of nSLN metastasis in patients with cT1/2N0 stage, undergoing TM and 1–2 SLN macrometastases under specific conditions, which will facilitate clinical identification of low-risk patients with nSLN metastasis, so as to guide the treatment decision whether to exempt ALND or ART.

## Methods

### Patients and data collection

Clinicopathological data, including age, BMI, total number of SLN, number of negative SLN, ratio of SLN macrometastases, preoperative neutrophil lymphocyte ratio (NLR), tumor size (TS), lymphovascular invasion (LVI), histological grade, human epidermal growth factor receptor-2 (HER2) status, estrogen receptor (ER) status, progesterone receptor (PR) status, Ki-67 index, pathological type, multifocal lesions, extracapsular invasion of SLN, molecular type, tumor site, family history of malignancy and menstrual status, of patients with invasive breast cancer who underwent up-front surgery with TM and SLNB at the People’s Hospital of Guangxi Zhuang Autonomous Region and the Fourth Affiliated Hospital of Guangxi Medical University from January 2017 to February 2022 were retrospectively analyzed. SLN-positive patients underwent further ALND.

### Inclusion and exclusion criteria

Inclusion criteria:

1. Diagnosis of invasive breast cancer, treated with TM and axillary SLNB;
2. TS ≤5 cm with cN0 (no palpable enlarged lymph nodes, no abnormal morphological and echoic lymph nodes were found on ultrasound) [12];
3. 1–2 macrometastatic SLN and receiving ALND.

Exclusion criteria:

1. SLN negative, SLN containing only isolated clusters tumor cells (ITCs) or micrometastases, and ≥3 SLN containing macrometastases found intraoperatively;
2. Receiving neoadjuvant therapy;
3. Incomplete clinicopathological data.

### SLNB and ALND procedure

After anesthesia and disinfection, 2 ml of 1% methylene blue was taken and injected subcutaneously into the areola 5–10 min before surgery. Blue-stained lymph nodes and their adjacent enlarged non-blue-stained lymph nodes were searched along the blue-stained lymphatic vessels as SLN and subjected to intraoperative frozen pathological diagnosis. SLN-positive patients underwent further ALND treatment. Axillary lymph nodes were routinely dissected at level I to II, and some patients dissected to level III. All SLNB and ALND procedures were completed prior to adjuvant chemotherapy.

### Judgment criteria

Histological grade [13]: The modified Scarff-Bloom-Richardson grading system was adopted: three indicators were evaluated independently and given a score of 1–3 based on the ratio of glandular duct formation, cellular atypia, and mitotic count. The histological grade of invasive carcinoma was classified into grade I (3–5 points), grade II (6–7 points), and grade III (8–9 points) according to the combined total score.

Tumor size: The primary outcome was the size of the pathological presentation (gross and microscopic measurements) of the primary tumor, combined with imaging modalities such as mammography, ultrasound, and MR imaging. For patients with intraductal carcinoma, the size of the invasion was the criterion.

Types of SLN metastasis: According to the American Joint Committee on Cancer (AJCC), ITCs are defined as small clusters of cells ≤0.2 mm in largest dimension, or single tumor cells, or fewer than 200 cells in a single histologic cross-section. Micrometastases are defined as tumor deposits >0.2 mm but ≤2.0 mm in largest dimension. Macrometastases are defined as tumor deposits >2.0 mm in largest dimension.

Ratio of SLN micrometastases: The ratio of the number of macrometastatic SLN to the total number of SLN.

### Statistical analysis

The SPSS 23 Statistics software was used to perform to determine risk factors. Chi-squared test was used for categorical data, while Student’s *T* test or Wilcoxon Rank Sum was employed for continuous measurement data. Multivariate unconditional logistic regression analysis was used for multivariate correlation analysis. The diagnostic accuracy was assessed by receiver operating characteristic (ROC) analysis. The area under the ROC curve (AUC) was used to evaluate the critical value of relevant factors. *P* < 0.05 was considered a statistically significant difference.

## Results

### Patients and treatments

**Fig. 1:**
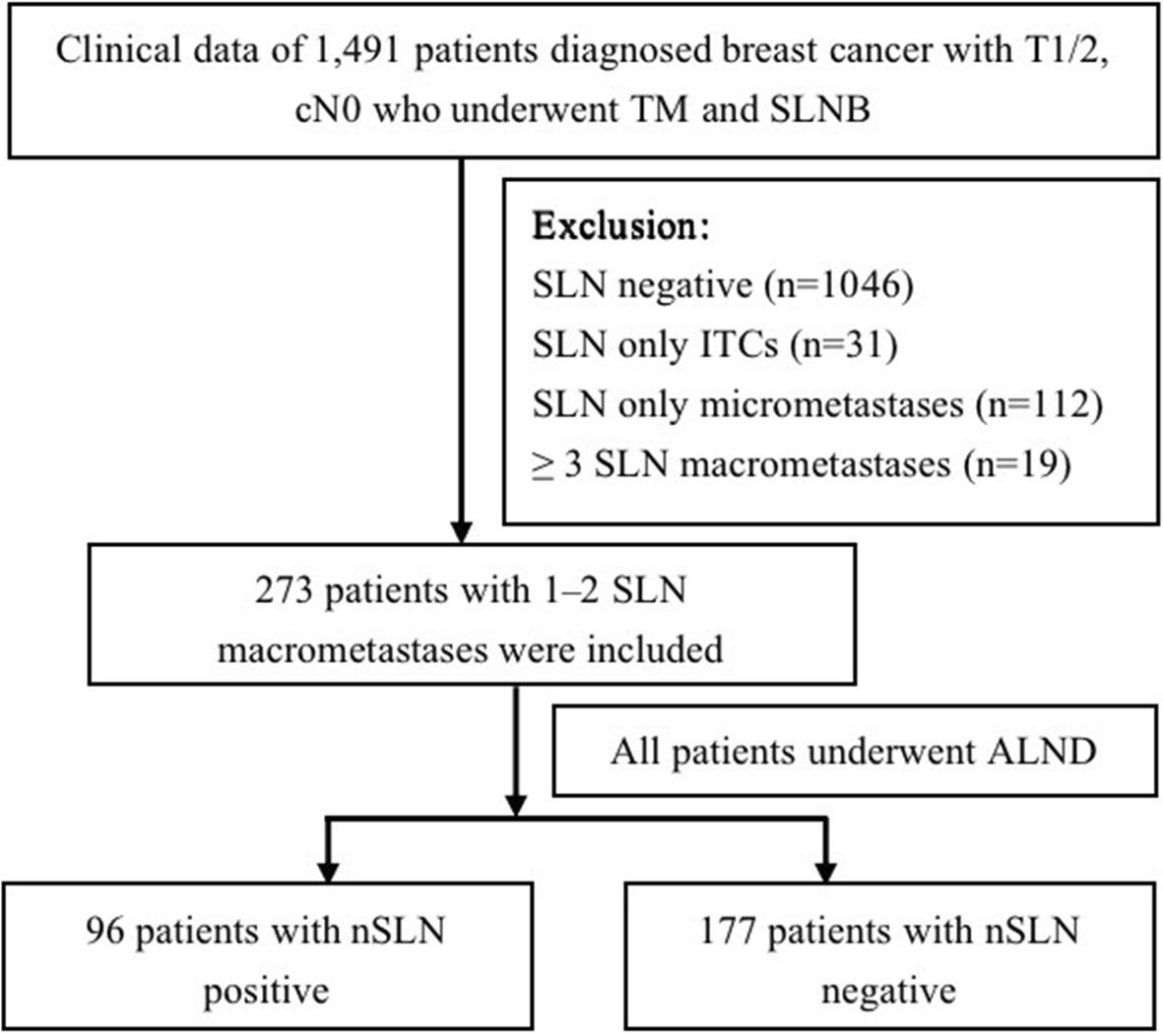
Research flow chart. TM, total mastectomy; SLNB, sentinel lymph node biopsy; ITCs, isolated tumor cell clusters; SLN, sentinel lymph node, ALND, axillary lymph node dissection; nSLN, non-sentinel lymph node

A total of 1491 patients with invasive breast cancer were treated with TM and SLNB, all of whom were female. Among them, patients with SLN 1–2 macrometastases accounted for 18.3% (*n* = 273), with a mean age of 51.5 years. Pathological data after ALND showed that a total of 35.2% (*n* = 96) patients developed nSLN metastasis (Fig. 1).

### Univariate and multivariate analyses

The comparison of clinicopathological factors showed significant differences in the total number of SLN, number of negative SLN, ratio of SLN macrometastases, TS, LVI, pathological type and tumor site between the nSLN-positive and nSLN-negative patients (*P* < 0.05), while the difference between the two groups in age, BMI, NLR, histological grade, molecular type, ER status, PR status, Ki-67 index, HER2 status, multifocal lesions, menstrual status, and family history of malignancy was not significant (Table 1). Extracapsular invasion of SLN was not found in either group and therefore was not included in the statistical analysis.

**Table 1:** Relationship between nSLN and clinicopathological factors

Characteristic	nSLN-negative (*n* = 177)	nSLN-positive (*n* = 96)	*t*/*z*/*x*^2^	*P* value
Age at diagnosis (year) (mean ± SD)	51.24 ± 10.44	51.89 ± 11.93	−0.466	0.642
BMI (kg/m^2^) (median (p25–p75))	23.31 (21.24–25.89)	23.12 (21.40–26.43)	−0.707	0.480
TS (mm) (median (p25–p75))	20 (15–25)	25 (20–30)	−3.429	0.001
Total number of SLN (median (p25–p75))	4 (3–5)	3 (2–4)	−2.937	0.003
Number of negative SLN (median (p25–p75) )	2.00 (1.00–4.00)	1.50 (0.25–3.00)	−3.959	0.001
Ratio of SLN macrometastases (median (p25–p75))	0.33 (0.25–0.50)	0.50 (0.33–0.92)	−4.987	0.001
NLR (median (p25–p75))	1.99 (1.61–2.68)	2.00 (1.53–2.67)	−0.803	0.423
Histological grade			6.428	0.093
I	20 (11.3)	3 (3.1)		
II	113 (63.8)	63 (65.6)		
III	34 (19.2)	21 (21.9)		
Unknown	10 (5.6)	9 (9.4)		
Pathological type			4.164	0.041
Invasive ductal carcinoma	172 (97.2)	88 (91.7)		
Others	5 (2.8)	8 (8.3)		
LVI			6.893	0.009
Negative	119 (67.2)	49 (51.0)		
Positive	58 (32.8)	47 (49.0)		
ER status			0.092	0.761
<10%	34 (19.2)	17 (17.7)		
≥10%	143 (80.8)	79 (82.3)		
PR status			0.026	0.872
<10%	57 (32.2)	30 (31.3)		
≥10%	120 (67.8)	66 (68.8)		
Ki-67 index			1.660	0.436
<14%	52 (29.4)	24 (25.0)		
≥14%, ≤30%	67 (37.9)	44 (45.8)		
>30%	58 (32.8)	28 (29.2)		
Her2 status			0.180	0.672
Negative	135 (76.3)	71 (74.0)		
Positive	42 (23.7)	25 (26.0)		
Molecular type			3.426	0.489
Luminal A	65 (36.7)	27 (28.1)		
Luminal B-Her (−)	58 (32.8)	36 (37.5)		
Luminal B-Her2 (+)	22 (12.4)	17 (17.7)		
ERBB2 (+)	15 (8.5)	6 (6.3)		
Basal-like	17 (9.6)	10 (10.4)		
Tumor site			11.353	0.023
Upper outer	80 (45.2)	61 (63.5)		
Lower outer	22 (12.4)	13 (13.7)		
Lower inner	15 (8.5)	3 (3.2)		
Upper inner	45 (25.4)	13 (13.7)		
Central portion	15 (8.5)	6 (6.3)		
Multifocal lesions			2.513	0.113
No	169 (95.5)	87 (90.6)		
Yes	8 (4.5)	9 (9.4)		
Menstrual status			1.887	0.170
Premenopausal	102 (57.6)	47 (49.0)		
Postmenopausal	75 (42.4)	49 (51.0)		
Family history of malignancy			3.227	0.196
None	133 (75.1)	71 (74.0)		
Breast or ovarian cancer	16 (9.0)	4 (4.2)		
Other malignant tumors	28 (15.8)	21 (21.9)		

The clinicopathological factors of the total number of SLN, number of negative SLN, ratio of SLN macrometastases, TS, LVI, pathological types, and tumor sites were included in multivariate analysis and indicated that TS (*P* = 0.002; OR: 1.051; 95% CI: 1.019–1.084) and ratio of SLN macrometastases (*P* = 0.0001; OR: 12.597; 95% CI: 4.302–36.890) were the independent risk factors for nSLN metastasis in breast cancer with 1–2 SLN macrometastases that underwent TM (Table 2).

**Table 2:** Multivariate analysis of clinicopathological factors for nSLN metastasis

Characteristic	OR	95% CI	*P* value
TS (mm)	1.051	1.019–1.084	0.002
Ratio of SLN macrometastases	12.597	4.302–36.890	0.0001
Tumor sites			0.054
Upper outer	1		Reference
Lower outer	0.777	0.344–1.754	0.544
Lower inner	0.255	0.066–0.980	0.047
Upper inner	0.405	0.193–0.853	0.017
Central portion	0.468	0.159–1.377	0.168

### ROC analysis of TS and ratio of SLN macrometastases

**Fig. 2:**
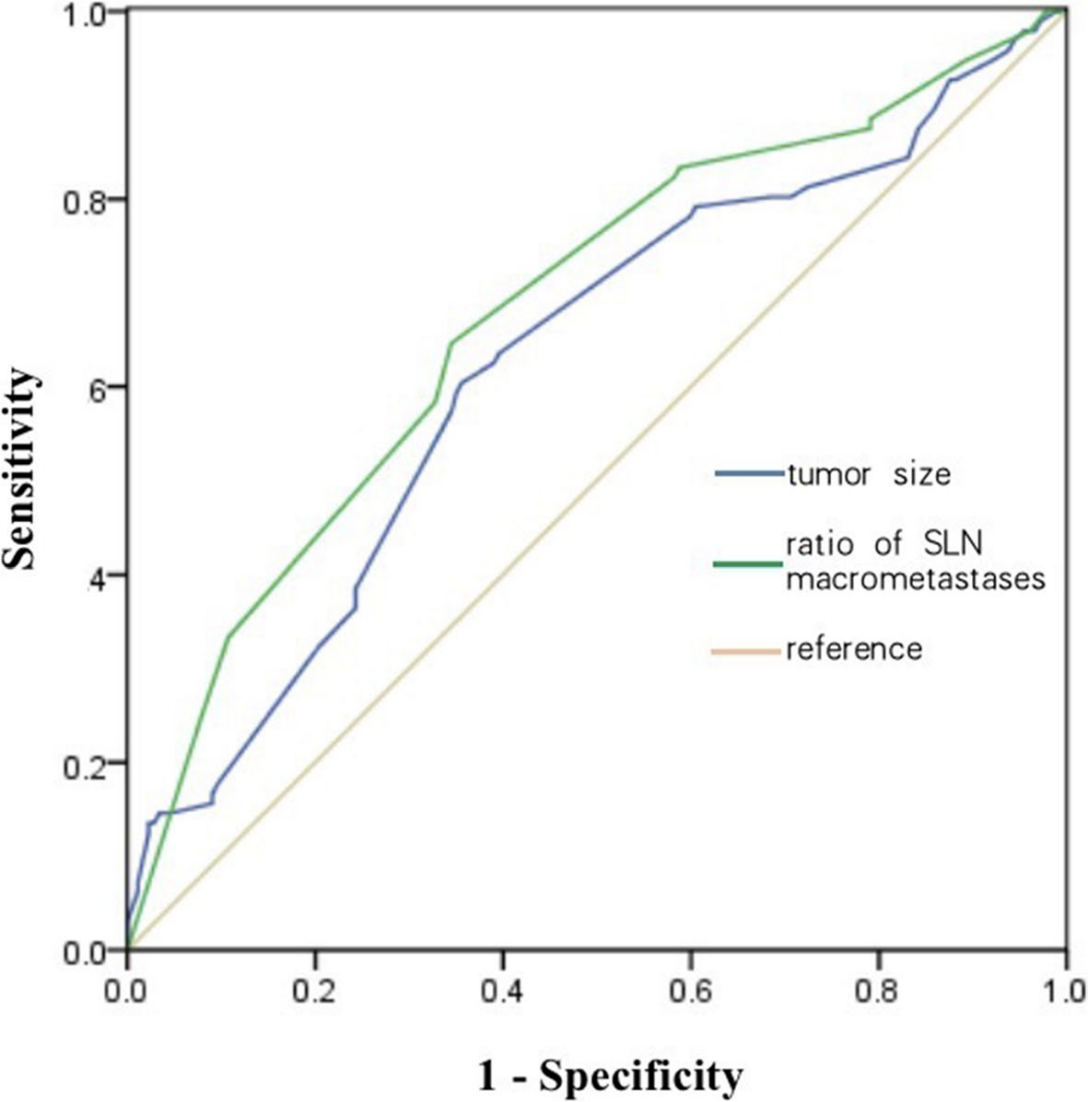
Receiver operating characteristic (ROC) curve. (Blue line) tumor size, the ROC analysis identified a cut-off point of 22 mm (AUC: 0.625, sensitivity: 60.4%, specificity: 64.4%); (green line) ratio of SLN micrometastases, the ROC analysis identified a cut-off point of 0.33 (AUC: 0.680, sensitivity: 64.6%, specificity: 65.5%)

**Fig. 3:**
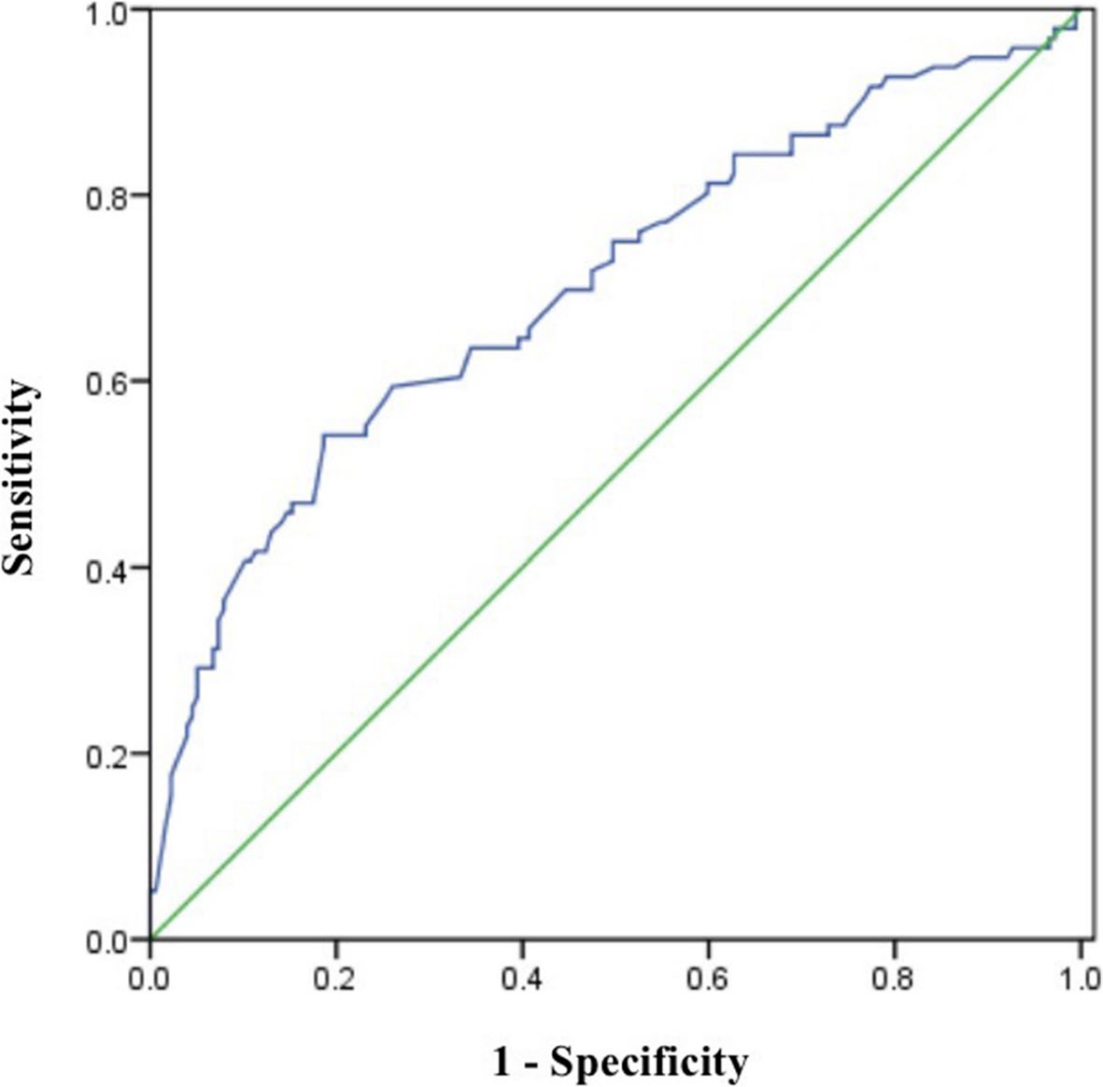
Receiver operating characteristic (ROC) curve of tumor size combined with ratio of SLN micrometastases (AUC: 0.700, sensitivity: 54.2%, specificity: 81.4%)

The cut-off points of TS and ratio of SLN macrometastases determined by receiver operating characteristic (ROC) analysis were 22 mm (AUC 0.625, sensitivity 60.4%, specificity 64.4%, *P* < 0.001, Fig. 2) and 0.33 (AUC 0.680, sensitivity 64.6%, specificity 65.5%, *P* < 0.001, Fig. 2), respectively. Combined with tumor size and ratio of SLN macrometastases, the ROC analysis showed AUC: 0.700, sensitivity: 54.2%, and specificity: 81.4% (Fig. 3). When TS >22 mm and ratio of SLN macrometastases >0.33, the incidence of nSLN metastasis was 73.6%, and when TS ≤22 mm and ratio of SLN macrometastases ≤0.33, the incidence of nSLN metastasis decreased to 17.1% (Table 3).

**Table 3:** Relationship between tumor size, ratio of SLN macrometastases, and incidence of nSLN metastasis

Characteristic	Amount	Incidence of nSLN metastasis
TS >22 mm and ratio of SLN macrometastases >0.33	53	73.6%
TS >22 mm and ratio of SLN macrometastases ≤0.33	68	27.9%
TS ≤22 mm and ratio of SLN macrometastases >0.33	70	32.9%
TS ≤22 mm and ratio of SLN macrometastases ≤0.33	82	17.1%
In total	273	35.2%

## Discussion

Surgery still has an irreplaceable clinical position in the comprehensive treatment of breast cancer. With the gradual change of the treatment concept, the principle of surgical treatment has shifted from maximally tolerable treatment to minimally effective treatment; therefore, the SLNB technique has become a routine axillary treatment for patients with clinically node-negative breast cancer. In patients with SLN-positive breast cancer, ALND remains the primary treatment for a long time [14]. However, 34.3% to 77.3% of patients with SLN-positive were found to have no nSLN metastasis after ALND [6, 8, 12]. With the publication of the follow-up results from the ACOSOG Z0011 trial [3], the AATRM 048/13/2000 trial [15] and IBCSG 23-01 trial [4], breast cancer patients with 1–2 SLN micrometastases for TM and 1–2 SLN micro/macrometastases for BCT can safely avoid ALND. However, whether patients with TM and 1–2 SLN macrometastases are exempted from ALND has not been adequately studied clinically. It is worth noting that there are still some studies that show a significant increase in recurrence and mortality rates associated with ALND omission after a significant amount of time [16, 17]. The EORTC AMAROS study suggested that ALND and ART after a positive SLN both provide good axillary control for T1/2 breast cancer. In addition, the ART can reduce the incidence of upper arm lymphedema by nearly 50% compared with ALND. However, it is important to note that the rate of arm lymphedema symptoms was still as high as 11% after 5 years of ART [5]. In this study, 64.8% of patients with TM and 1–2 SLN macrometastases did not develop nSLN metastases, implying that they did not benefit from undergoing ALND surgery. It is important to investigate the risk factors associated with nSLN metastasis in patients with TM and 1–2 SLN macrometastases and to screen patients who can avoid ALND or ART, which can reduce the trauma caused by surgery or radiotherapy.

Little research has concentrated on the circumstances of TM with SLN macrometastases, despite the fact that numerous studies have previously examined the risk factors for SLN-positive and nSLN metastasis in breast cancer. Previous studies have found that the size of SLN metastases, extracapsular invasion of SLN, the number of positive and negative SLN, the ratio of positive SLN, TS, LVI, histological grade, NLR, and other clinicopathological factors were all related to the occurrence of nSLN metastasis [6–11]. It is important to note that the type of surgery, TM or breast-conserving surgery, was not a factor in nSLN metastasis. However, it may affect the choice of adjuvant therapy, such as the indication of radiotherapy, then may affect the prognosis of patients. In the analysis of our group, under the conditions of cT1/2N0 and TM with 1–2 SLN macrometastases, we found that the total number of SLN, number of negative SLN, ratio of SLN macrometastases, TS, LVI, pathological type, and tumor site were correlated with the occurrence of nSLN metastasis. Multivariate analysis further found that TS and ratio of SLN macrometastases were independent predictors of nSLN metastasis. When TS >22 mm and ratio of SLN macrometastases >0.33, the incidence of nSLN metastasis was 73.6%, and when TS ≤22 mm and ratio of SLN macrometastases ≤ 0.33, the incidence of nSLN metastasis decreased to 17.1%. It is suggested that selecting a small tumor burden before surgery and increasing the number of SLN detected during surgery can improve the accuracy of negative prediction of nSLN. The findings are currently insufficient to employ the aforementioned characteristics as indicators of whether to perform ALND; however, in clinical practice, they have important reference value for case selection.

Although ALND is currently recommended for patients with SLN-positive breast cancer undergoing TM, the real-world data on SLN-positive disease without ALND are equally noteworthy. In the entire study cohort, 4093 patients had T1/2N0M0 breast cancer with 1–2 SLN metastases and underwent TM. The incidence of ALND decreased from 89.9% in 2010 to 55.5% in 2015. Among them, the incidence decreased from 82% to 8% in patients with SLN micrometastases and from 93% to 63% in those with macrometastases. In multivariable analysis, factors associated with the omission of ALND were pT1 status, older age, more SLN removed, fewer positive SLN, and SLN micrometastasis [18]. In addition, in another study of 329 patients with 1–2 SLN metastases who underwent TM, 201 patients received ALND. Compared with patients who received SLNB alone, patients who received ALND were characterized by higher tumor grade and higher proportion of hormone receptor-negative, adjuvant radiotherapy was similar in both groups. After a median follow-up of 51 months, there were no significant differences in OS and local recurrence rates between the two groups [19]. Kim and his colleagues observed 3632 breast cancer patients who underwent TM with 1–2 SLN metastases, of which 883 patients did not receive ALND. Compared with the ALND group, the axillary lymph node tumor load was lower and the proportion of patients receiving adjuvant chemotherapy was higher. However, no statistical analysis was performed on the receiving of adjuvant radiotherapy in both groups. There was no difference in OS between the two groups at a median follow-up of 54 months [20]. Axillary recurrence (AR) is the focus of long-term follow-up of all breast cancer patients undergoing SLNB. Previous studies have found that axillary recurrence is associated with a variety of factors, including the grade 2 or 3 disease, the absence of radiotherapy and molecular type [21]. The rate of AR after SLNB is usually low, as Francissen reported that after a median of 45 months of follow-up, the AR rate of 3268 patients from 16 studies who had SLN macrometastases but did not receive ALND was only 0.7% [22]. All the above data suggest that avoiding ALND in breast cancer patients with TM and 1–2 SLN metastases appears to be safe when selecting appropriate low-risk cases.

Several prospective controlled studies have been performed to show whether patients with 1–2 SLN macrometastases treated with TM can be exempted from axillary treatment. Among them, the SINODAR ONE study was a prospective non-inferiority multicenter randomized study aimed at assessing the role of ALND in patients underwent either BCT or TM for T1–2 breast cancer with 1–2 SLN macrometastases. Among 879 patients, there were no significant differences in OS or RFS between ALND recipients and non-ALND recipients at a mean follow-up of 32 months [23]. However, the low proportion of patients who underwent TM (24.8%) and the short follow-up time in this study still cannot answer alone the question of whether breast cancer patients who underwent TM with 1–2 SLN macrometastases can be exempted from ALND. In addition, SENOMAC and POSNOC studies are still in progress. The SENOMAC study, a non-inferiority study, primarily follows the inclusion criteria of the ACOSOG Z0011 study, but allows the inclusion of T1–3 patients who underwent TM. A total of 3500 patients are randomized 1:1 to either undergo ALND or not, and the primary endpoint is breast cancer-specific survival at 5 years [24]. The POSNOC study is a randomized, multi-center, non-inferiority trial designed to evaluate whether breast cancer patients with T1–2, 1–2 SLN macrometastases who underwent TM or BCT without axillary treatment are non-inferior to ALND or ART. The sample size is 1900 and participants will be followed up for 5 years. The primary endpoint is axillary recurrence at 5 years. As of June 2021, POSNOC study had completed 1866 cases [25]. The results of the ACOSOG Z0011 and IBCSG 23-01 studies have changed the previous breast cancer treatment strategy. We expect the new research results will lead to new treatment decisions. In the recent-published SENOMAC study of health-related quality of life and arm morbidity after axillary surgery, 976 patients (501 in SLNB and 475 in ALND) were enrolled and it was found that arm morbidity was significantly worse affected by ALND than by SLNB 1 year after surgery. The results underline the importance of ongoing attempts to safely de-escalate axillary surgery [26].

This was a small retrospective study from two clinical centers, and the insufficient number of cases may have influenced the analysis of the results. The subjects in this study were the breast cancer patients with T1/2, cN0, and TM with 1–2 SLN macrometastases, and the study comprehensively evaluated the influence of different clinicopathological factors on nSLN metastasis and further analyzed the role of related influencing factors. This has implications for the choice of whether to waive ALND in clinical practice. However, there were some uncontrolled research biases in this study, which may have a certain impact on the results. We look forward to more randomized controlled studies to evaluate the safety of ALND exclusion in breast cancer patients with T1/2, cN0, and TM with 1–2 SLN macrometastases. In addition, due to limited access to nuclides, methylene blue dye was used in this study as a single agent in SLNB for the treatment of early breast cancer. Although the accuracy and identification rate of methylene blue dye used in SLNB have been widely verified [27], it could still raise concerns about the rise of false negative rates.

## Conclusions

The breast cancer patients with cT1/2N0 stage, who underwent TM and 1–2 SLN macrometastases, and the occurrence of nSLN metastasis is closely related to TS and the ratio of SLN macrometastases. When the TS ≤22 mm and macrometastatic SLN does not exceed 1/3 of the total number of detected SLN, the incidence of nSLN metastasis is significantly reduced, but whether ALND can be exempted needs further exploration.

## Data Availability

The datasets used and/or analyzed during the current study are available from the corresponding author on reasonable request.

## Abbreviations

ALND: Axillary lymph node dissection
AR: Axillary recurrence
ART: Axillary radiotherapy
AUC: The area under the ROC curve
BTC: Breast-conserving therapy
ER: Estrogen receptor
HER2: Human epidermal growth factor receptor-2
ITCs: Isolated tumor cell clusters
LVI: Lymphovascular invasion
NLR: Neutrophil lymphocyte ratio
nSLN: Non-sentinel lymph node
PR: Progesterone receptor
ROC: Receiver operating characteristic
SLN: Sentinel lymph node
SLNB: Sentinel lymph node biopsy
TM: Total mastectomy
TS: Tumor size
